# Associations between co‑exposure to heavy metals and vertebral compression fracture, as well as femoral neck bone mineral density: A cross-sectional study from NHANES data

**DOI:** 10.1371/journal.pone.0303418

**Published:** 2024-05-22

**Authors:** Xurong Yang, Li Li, Lixiong Nie

**Affiliations:** 1 Department of Orthopedic Surgery, Jiangle General Hospital of FuJian Province, Sanming, China; 2 Department of Endocrinology, Jiangle General Hospital of FuJian Province, Sanming, China; 3 Department of Critical Care Medicine, Jiangle General Hospital of FuJian Province, Sanming, China; Al Mansour University College-Baghdad-Iraq, IRAQ

## Abstract

**Objective:**

Accumulating evidence showed that exposure to heavy metals was harmful to human health. Little is known regarding the mixing effects of multiple metal exposures on vertebral compression fracture (VCF) and femoral neck bone mineral density (BMD). This study aimed to explore the individual and joint effects of four heavy metals [manganese (Mn), lead (Pb), cadmium (Cd) and mercury (Hg)] on VCF risk and femoral neck BMD.

**Methods:**

This cross-sectional study included 1,007 eligible individuals with vertebral fractures from National Health and Nutrition Examination Survey 2013–2014. The outcome was the risk of VCF and femoral neck BMD. Weighted multivariate logistic regression was used to explore the individual effect of four heavy metals on the VCF risk, separately. Weighted multivariate linear regression was used to explore the individual effect of four heavy metals on the femoral neck BMD, separately. Adopted bayesian kernel machine regression (BKMR) model and quantile-based g computation (qgcomp) to examine the joint effects of four heavy metals on the VCF risk and femoral neck BMD.

**Results:**

Among the population, 57 individuals developed VCF. After adjusting covariates, we found no statistical differences regarding the individual effects of four heavy metals on the risk of VCF. BKMR model and qgcomp indicated that there were no statistical differences regarding the joint effects between four heavy metals on the VCF risk. In addition, we found that Cd was associated with femoral neck BMD, and an increase in the mixture of heavy metal exposures was associated with a decreased risk of femoral neck BMD.

**Conclusion:**

No significant correlation was observed between co-exposure to Mn, Pb, Cd and Hg and VCF risk. But co-exposure to Mn, Pb, Cd and Hg may be associated with femoral neck BMD.

## Introduction

Vertebral compression fracture (VCF) is a type of fracture in middle-aged and elderly population, and its incidence increases with age [1]. VCF was associated with the risk of osteoporosis, affecting 700,000 cases each year in the United States [2]. VCF causes chronic back and neck pain, reduced lung volumes, impaired quality of life and increased mortality [2, 3]. Previous studies have indicated that VCF may increase the risk of subsequent vertebral and non-vertebral fractures [4, 5]. Therefore, early implementation of appropriate prevention is important to reduce the risk of VCF, improve quality of life and increase life expectancy.

Heavy metals are commonly found in the surrounding air, soil, food and water [6]. Chronic exposures to the heavy metals may affect human health, such as female reproductive health [7], bone mineral density (BMD) loss [8], and cancer [9]. A meta-analysis conducted by Cheng X et al., reported that high cadmium (Cd) exposure may constitute a potential risk factor for fractures of any nature [10]. Wang C, et al., found that people exposed to manganese (Mn) may be a risk factor of osteopenia in a cross-sectional study with 9,732 subjects [11]. Most existing studies have only focused on the relationship between individual metal exposures and bone health [10, 11]. However, heavy metals are often co-present in the environment, and people are more likely to be exposed to multiple exposures at the same time [12]. Nowadays, a study with 2,545 United States adults suggested that multiple metals exposure was associated with reduced BMD by using weighted quantile sum and bayesian kernel machine regression (BKMR) models [13]. To the best of our knowledge, little is known about the mixing effects of multiple metal exposures on VCF risk or femoral neck BMD.

Herein, in the present study, we aimed to explore the individual and joint effects of four heavy metals [Mn, lead (Pb), Cd and mercury (Hg)] on the VCF risk and femoral neck BMD. The findings may provide new evidence for the link between heavy metal exposure and VCF risk/ femoral neck BMD.

## Methods

### Study population

In this cross-sectional study, we analyzed data from National Health and Nutrition Examination Survey (NHANES) 2013–2014, the study year in which vertebral fractures were only assessed in participants aged ≥40 years. NHANES database is a representative cross-sectional survey of non-institutionalized U.S. population. The survey examines approximately 5,000 people each year. These people are located in counties throughout the country, and 15 of which are visited annually [14]. (https://www.cdc.gov/nchs/nhanes/about_nhanes.htm). The requirement of ethical approval for this was waived by the Institutional Review Board of Jiangle General Hospital of FuJian Province, because the data was accessed from NHANES (a publicly available database). The need for written informed consent was waived by the Institutional Review Board of Jiangle General Hospital of FuJian Province due to retrospective nature of the study.

**Fig 1:**
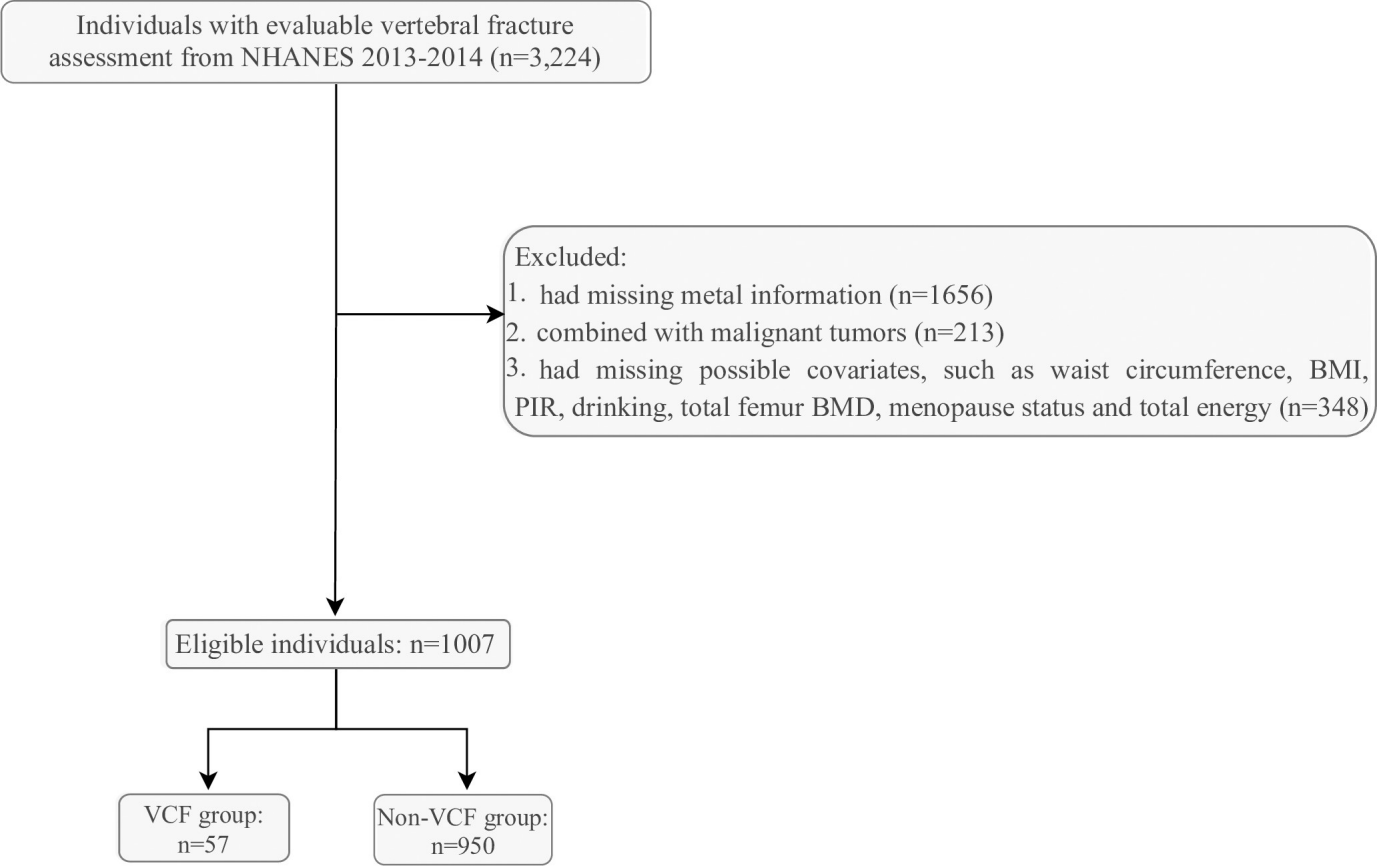
The process of participants selection.

3,224 individuals aged ≥40 years who had the evaluable vertebral fracture assessment (VFA) were included [14] (https://wwwn.cdc.gov/Nchs/Nhanes/2013-2014/DXXVFA_H.htm#DXXVFAST). Then, we further excluded some individuals who (1) had missing metal information, (2) combined with malignant tumors, (3) had missing possible covariates, such as waist circumference, body mass index (BMI) and poverty-to-income ratio (PIR). Finally, 1,007 eligible individuals were included in the current study. Fig 1 shows the process of participants selection.

### Exposures: Measurement of heavy metal

For individuals enrolled in the NHANES database, whole blood specimens were collected. The concentrations of Mn, Pb, Cd and Hg in whole blood samples were measured directly by using mass spectrometry. The lower limit of detection (LLOD) for Mn, Pb, Cd and Hg was 0.99 μg/L, 0.07 ug/dL, 0.10 μg/L and 0.28 μg/L, respectively. For values below LOD, it would be calculated as the LLOD divided by the square root of 2.

(https://wwwn.cdc.gov/Nchs/Nhanes/2013-2014/PBCD_H.htm#Analytic_Notes).

### Outcomes: Assessment of vertebral compression fracture and femoral neck BMD

The primary outcome of this study was whether VCF occurred. For NHANES individuals, VFA is obtained by utilizing dual energy x-ray absorptiometry (DXA) to conduct a lateral scan of the thoraco-lumbar spine. The DXA examinations were conducted by trained and certified radiology technologists. All scans were analyzed by Optasia Spinalizer software using Genant’s semiquantitative technique. Each vertebra was graded as normal (Grade 0), mild (Grade 1), moderate (Grade 2), or severe (Grade 3) fracture [15]. Grade 1 and above were diagnosed as VCF [16, 17]. (https://wwwn.cdc.gov/Nchs/Nhanes/2013-2014/DXXVFA_H.htm). The secondary outcome was femoral neck BMD. Femoral neck BMD was measured by DXA. The DXA examinations were conducted by certified radiologic technologists using Hologic QDR-4500A fan-beam densitometers (Hologic; Bedford, MA), and the data analysis was performed utilizing the Hologic APEX software, version 4.0. (https://wwwn.cdc.gov/Nchs/Nhanes/2013-2014/DXXFEM_H.htm).

### Data collection

We extracted some data of all eligible individuals from the NHANES 2013–2014: age, gender, race/ethnicity, educational level, marital status, waist circumference (cm), BMI (kg/m^2^), PIR, smoking, drinking, physical activity [metabolic equivalent (MET)·min/week], previous fracture, parental fracture, history of glucocorticoid use, history of anti-osteoporosis medication use, diabetes, hypertension, total femur BMD (g/cm^2^), femoral neck BMD (g/cm^2^), menopause status, total energy (kcal), total calcium intake (mg), total vitamin D intake (mcg), Mn (ug/L), Pb (ug/dL), Cd (ug/L) and Hg (ug/L). Physical activity was considered as energy consumption, which was calculated as the number of minutes of activity per week multiplied by the MET score for each activity and summed [18]. The intake of total energy/total calcium intake/total vitamin D intake was calculated by adding up the dietary and supplemental intake in this study.

### Statistical analysis

In descriptive analysis, continuous variables were described by mean and standard error (S.E), and weighted t-test was used for comparison between two groups. Categorical variables were described by the number of cases and constituent ratio [n (%)], and χ^2^ test was performed to compare the difference between groups. In the weighted univariate logistic regression analysis, we screened some possible confounding factors related to VCF (*P*<0.05, S1 Table). A weighted univariate linear regression model was established to identify the confounding factors potentially associated with femoral neck BMD (*P*<0.05, S1 Table). Then, our study was conducted in two phases. The first phase was to explore the individual effect of four heavy metals on the VCF risk and femoral neck BMD, separately by weighted multivariate logistic regression. Odds ratio (OR) with a 95% confidence interval (CI) was calculated. Weighted multivariate linear regression analysis was used to assess the individual effect of four heavy metals on the femoral neck BMD, separately. The second phase was to examine the joint effects of four heavy metals on the VCF risk and femoral neck BMD by BKMR model and quantile-based g computation (qgcomp). *P*<0.05 was considered as statistically significant difference.

### Bayesian kernel machine regression model

BKMR is a semi-parametric method to assess the nonlinear and/or interaction associations of exposure-outcome [19]. BKMR model underwent 10,000 iterations using Markov chain Monte Carlo algorithm. Individual metal exposures were grouped using Pearson correlation analysis (S1 Fig) [20], and we found higher correlation for Pb and Cd compared to correlations for other metal exposures. Pb and Cd were classified as group 1, while Mn and Hg were classified as group 2. The group posterior inclusion probability (Group PIP) and conditional posterior inclusion probability (Cond PIP) were calculated to select the key metals that had an impact on VCF. The combined effect on VCF was calculated by comparing mixed heavy metals at or above the 60th percentile to the 50th percentile. The exposure-response relationship for each heavy metal and VCF risk were explored by taking all other metabolites at median levels. The interaction associations were assessed by taking exposure 1 heavy metal at its 25%, 50%, and 75% levels and other metabolites fixed at their median levels. R bkmr package was used for this analysis.

### Quantile-based g computation

qgcomp is a parameterized model based on g-computation to for evaluating the effect of increasing all exposures in the mixture [21]. In this study, we adopted Bootstrapping method to calculate the population average exposure effect. R qgcomp package was used for this analysis.

## Results

### Characteristics of participants

Table 1 shows the characteristics of all eligible individuals (n = 1007). The average age was 55.98 (0.24) years. 48.67% were female. Of these participants, 6.77% are Mexican American, 71.50% are non-Hispanic white, 10.82% are non-Hispanic black, 4.18% are other Hispanic, and 6.73% are other races. In addition, among the population, 57 individuals developed VCF. We compared the differences in characteristics between VCF group (n = 57) and non-VCF group (n = 950). Compared with the non-VCF group, individuals with VCF were older (*P*<0.001). Individuals with VCF showed lower BMI, total femur BMD and femoral neck BMD than individuals with non-VCF (*P*<0.05).

**Table 1:** The characteristics of all eligible individuals.

Variables	Total (n = 1007)	Non-VCF group (n = 950)	VCF group (n = 57)	*P*
Age, year, Mean (S.E)	55.98 (0.24)	55.49 (0.28)	64.59 (1.98)	<0.001
Gender, female, n (%)	495 (48.67)	465 (48.32)	30 (54.97)	0.468
Race/ethnicity, n (%)				0.252
Mexican American	128 (6.77)	124 (6.96)	4 (3.25)	
Other Hispanic	88 (4.18)	84 (4.23)	4 (3.17)	
Non-Hispanic White	451 (71.50)	414 (70.92)	37 (81.83)	
Non-Hispanic Black	203 (10.82)	195 (10.96)	8 (8.31)	
Other Race—Including Multi-Racial	137 (6.73)	133 (6.92)	4 (3.44)	
Education, n (%)				0.764
Less than 9th grade	83 (4.80)	79 (4.82)	4 (4.46)	
9-11th grade (Includes 12th grade with no diploma)	118 (8.82)	111 (8.70)	7 (11.03)	
High school graduate/ GED or Equivalent	234 (22.60)	216 (22.16)	18 (30.52)	
Some College or AA degree	289 (29.29)	273 (29.44)	16 (26.67)	
College Graduate or above	283 (34.49)	271 (34.89)	12 (27.32)	
Marital status, n (%)				0.003
Married	595 (65.27)	571 (66.44)	24 (44.33)	
Not Married	412 (34.73)	379 (33.56)	33 (55.67)	
Waist circumference, cm, Mean (S.E)	100.54 (0.48)	100.67 (0.47)	98.25 (2.97)	0.427
BMI, kg/m^2^, Mean (S.E)	28.92 (0.22)	29.05 (0.21)	26.63 (0.92)	0.015
PIR, ratio, Mean (S.E)	3.22 (0.13)	3.25 (0.13)	2.75 (0.30)	0.064
Smoking, n (%)				0.381
Yes	174 (15.01)	165 (15.26)	9 (10.64)	
No	549 (54.77)	521 (55.11)	28 (48.75)	
Quit smoking	284 (30.22)	264 (29.63)	20 (40.62)	
Drinking, n (%)				0.013
Yes	738 (80.80)	699 (81.32)	39 (71.43)	
No	136 (10.02)	128 (10.07)	8 (9.06)	
Abstinence from alcohol	133 (9.18)	123 (8.60)	10 (19.51)	
Physical activity, MET·min, Mean (S.E)	2926.58 (167.88)	2965.78 (169.85)	2227.50 (642.99)	0.284
Previous fracture, yes, n (%)	9 (0.62)	9 (0.66)	0 (0.00)	
Parental fracture, yes, n (%)	70 (7.92)	63 (7.71)	7 (11.64)	0.662
History of glucocorticoid use, yes, n (%)	61 (5.87)	55 (5.77)	6 (7.53)	0.492
History of anti-osteoporosis medication use, yes, n (%)	33 (3.21)	26 (2.57)	7 (14.51)	<0.001
Diabetes, yes, n (%)	362 (33.26)	337 (33.30)	25 (32.70)	0.941
Hypertension, yes, n (%)	579 (53.29)	534 (52.18)	45 (73.17)	0.064
Total femur BMD, g/cm^2^, Mean (S.E)	0.96 (0.01)	0.97 (0.01)	0.82 (0.02)	<0.001
Femoral neck BMD, g/cm^2^, Mean (S.E)	0.79 (0.01)	0.80 (0.01)	0.66 (0.02)	<0.001
Menopause status, n (%)				0.050
Yes	343 (33.61)	315 (32.70)	28 (49.97)	
No	152 (15.06)	150 (15.62)	2 (5.00)	
Not applicable (male)	512 (51.33)	485 (51.68)	27 (45.03)	
Energy, kcal, Mean (S.E)	2101.52 (39.09)	2116.01 (35.04)	1843.09 (213.99)	0.200
Calcium, mg/dL, Mean (S.E)	936.26 (19.90)	936.37 (19.92)	934.30 (85.72)	0.981
Vitamin D, mcg, Mean (S.E)	4.70 (0.19)	4.66 (0.21)	5.24 (0.49)	0.325
Total energy intake, kcal, Mean (S.E)	2106.30 (39.20)	2120.91 (35.18)	1845.65 (214.30)	0.197
Total calcium intake, mg/dL, Mean (S.E)	1110.88 (25.39)	1105.84 (24.57)	1200.83 (114.26)	0.412
Total Vitamin D intake, mcg, Mean (S.E)	17.56 (1.38)	17.68 (1.40)	15.29 (2.51)	0.300
Pb*, ug/dL, Mean (S.E)	0.13 (0.03)	0.12 (0.03)	0.23 (0.11)	0.321
Cd*, ug/L, Mean (S.E)	-1.15 (0.04)	-1.16 (0.04)	-0.97 (0.12)	0.162
Hg*, ug/L, Mean (S.E)	-0.07 (0.06)	-0.07 (0.06)	-0.10 (0.15)	0.854
Mn*, ug/L, Mean (S.E)	2.19 (0.01)	2.20 (0.01)	2.09 (0.09)	0.273

GED, general educational development; AA, associate of arts; BMI, body mass index; PIR, poverty-to-income ratio; MET, metabolic equivalent; BMD, bone mineral density; Pb, lead; Cd, cadmium; Hg, mercury; Mn, manganese; VCF, vertebral compression fracture; SE, standard error; * represents the level of metal exposure after ln conversion

### Individual effect of four heavy metals on the VCF risk

We adopted a weighted multivariate logistic regression to explore the individual effect of four heavy metals on the VCF risk, separately. As shown in Table 2, after adjusting age, marital status, BMI, history of anti-osteoporosis medication use, total femur BMD, femoral neck BMD and menopause status, we found no statistical differences regarding the individual effects of four heavy metals on the risk of VCF (*P*>0.05).

**Table 2:** The individual effect of four heavy metals on the VCF risk.

Variables	Model 1		Model 2	
	OR (95%CI)	*P*	OR (95%CI)	*P*
Pb*	1.19 (0.83–1.71)	0.314	0.84 (0.56–1.26)	0.378
Cd*	1.23 (0.93–1.61)	0.134	0.89 (0.63–1.25)	0.476
Hg*	0.97 (0.64–1.45)	0.856	1.00 (0.64–1.56)	0.984
Mn*	0.72 (0.37–1.40)	0.309	0.73 (0.37–1.47)	0.356

Pb, lead; Cd, cadmium; Hg, mercury; Mn, manganese; VCF, vertebral compression fracture; OR, odds ratio; CI, confidence interval; * represents the level of metal exposure after ln conversion

Model 1: did not adjust any variables

Model 2: adjusted for age, marital status, body mass index, history of anti-osteoporosis medication use, total femur bone mineral density, femoral neck bone mineral density and menopause status.

### Joint effects of four heavy metals on the VCF risk

**Fig 2:**
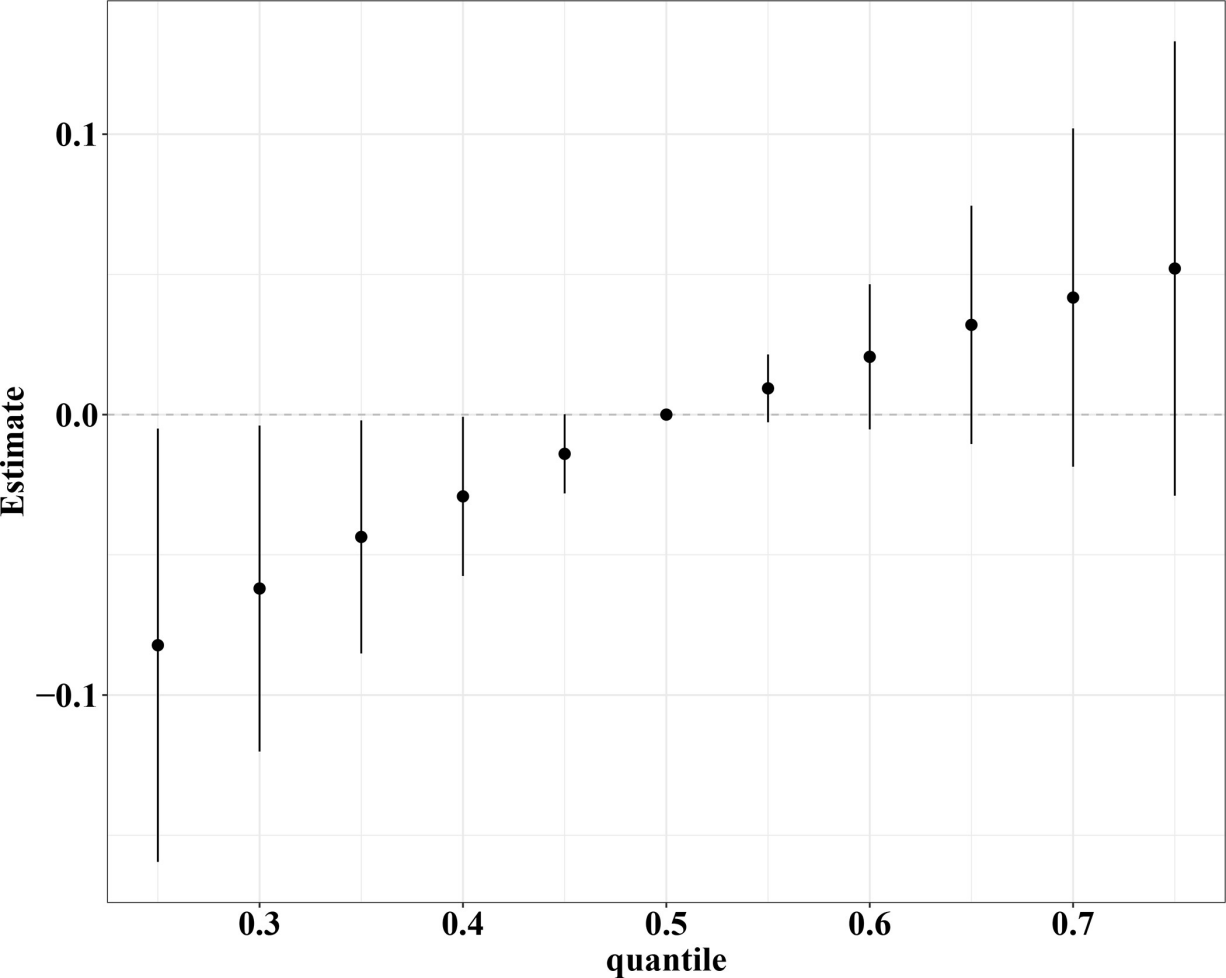
The overall association between heavy metal mixtures and VCF risk.

We further analyzed the joint effects of four heavy metals on the VCF risk by the BKMR model. Table 3 displays the Group posterior inclusion probability (Group PIP) and Conditional posterior inclusion probability (Cond PIP) obtained by the BKMR model. The Group PIP of group 2 was greater than group 1 (0.7444 *vs* 0.3592). In the group 2, the Cond PIP of Mn was greater than Hg (0.9154 *vs* 0.0846), which indicated that Mn contributed most to the model for the VCF risk. Subsequently, Fig 2 reveals the overall association between heavy metal mixtures and VCF risk. We observed that the ln-transformed VCF risk tended to increase when the blood mixed heavy metals at their 60th and above percentile compared to their 50th percentile. The result of exposure-response relationship indicated that a slightly inverse link between Mn and ln-transformed VCF risk (S2 Fig).

**Table 3:** The Group PIP and Cond PIP obtained by the BKMR model.

Variables	Group	Group PIP	Cond PIP
Pb	1	0.3592	0.6013
Cd	1	0.3592	0.3987
Hg	2	0.7444	0.0846
Mn	2	0.7444	0.9154

Group PIP, Group posterior inclusion probability; Cond PIP, Conditional posterior inclusion probability; BKMR, bayesian kernel machine regression; Pb, lead; Cd, cadmium; Hg, mercury; Mn, manganese; VCF, vertebral compression fracture

In addition, there might be a potential interaction between Pb and Mn for ln-transformed VCF risk (S3 Fig). Although there was no significant difference, we found that each additional quantile in the qgcomp index may be positively associated with the risk of VCF in the adjusted model (Table 4, S4 Fig).

**Table 4:** Qgcomp model to assess the joint effects of four heavy metals on the VCF risk.

Model	OR (95%CI)	*P*
Qgcomp	1.09 (0.85–1.39)	0.516

CI, confidence interval; OR, odds ratio; Qgcomp, quantile-based g computation

Qgcomp model adjusted for age, marital status, body mass index, history of anti-osteoporosis medication use, total femur bone mineral density, femoral neck bone mineral density and menopause status.

### Individual effects of four heavy metals on the femoral neck BMD

The present study additionally assessed the individual /joint effects of four heavy metals on the femoral neck BMD by using weighted univariate and multivariate linear models. As shown in S2 Table, after adjusting age, gender, race/ethnicity, educational level, marital status, drinking, parental fracture, BMI, waist circumference, history of glucocorticoid use, history of anti-osteoporosis medication use, diabetes, menopause status, and total energy, we found that Cd was associated with femoral neck BMD (β  =  -0.05, 95%CI: -0.03, -0.01, *P * =  0.043).

### Joint effects of four heavy metals on the femoral neck BMD

Using femoral neck BMD as the outcome, the mixed effect model BKMR for heavy metal exposure was established. In the fully adjusted BKMR model, a decreasing trend in the overall impact of four heavy metals on femoral neck BMD was observed, indicating that an increase in the mixture of heavy metal exposures was associated with a decreased risk of femoral neck BMD (S5 Fig). In the fully adjusted qgcomp model, the qgcomp index demonstrated a positive correlation with a reduction in the risk of femoral neck BMD (S3 Table, OR = 0.97, 95%CI: 0.95–0.98, *P* = 0.001). In the qgcomp model, Cd received the highest negative weights for the femoral neck BMD (S6 Fig).

## Discussion

In this study, we used some statistical methods to investigate the individual and joint effects of Mn, Pb, Cd and Hg on the VCF risk. There were no statistical differences regarding the individual effects of Mn, Pb, Cd and Hg on the risk of VCF by weighted multivariate logistic regression, and the joint effects on the VCF risk by BKMR and qgcomp model. In addition, we found that an increase in the mixture of heavy metal exposures was associated with a decreased risk of femoral neck BMD.

To our knowledge, there have been several reports concerning the effects of heavy metals on bone diseases. In the meta-analysis study of Jalili C, et al., they found that exposure to Cd and Pb may be linked with an increased risk of osteopenia or osteoporosis [21]. Previously, several studies have stated possible mechanisms for the effects of heavy metals on bone structure. For example, Cd may affect BMD by stimulating osteoclast differentiation and activity [22]. In addition, the harmful effects of Cd on bone metabolism may also include oxidative stress, autophagy, apoptosis and mitochondrial dysfunction [10, 23, 24]. Nevertheless, no significant association was observed between individual heavy metal with VCF risk in this analysis.

At present, more and more studies have begun to focus on the mixed effects of metal exposure on the risk of bone diseases. A cross-sectional study of 627 Chinese adults aged ≥50 years examined the relationship of co-exposure to Mn, iron (Fe), copper (Cu) and selenium (Se) and osteoporosis risk, and BKMR model showed that co-exposure to Mn, Fe, Cu, and Se was associated with reduced osteoporosis risk [12]. Not only that, a population-based study of United States adults indicated that co-exposure to Mn, Pb, Cd, Hg Cu, Se, and zinc (Zn) may be risk factor for BMD [13]. However, few studies have been conducted to investigate the relationship between mixed exposures of Mn, Pb, Cd and Hg and the risk of VCF. In the present study, we found that VCF risk tended to increase when the blood mixed heavy metals at their 60th and above percentile in the BKMR model. In other words, the greater the level of mixed heavy metals exposure, the higher the risk of VCF.

Not only that, this study also assessed the individual and joint effects of four heavy metals on the femoral neck BMD. Several prior studies have suggested a potential association between heavy metals and femoral neck BMD [13, 25, 26]. One animal study indicated that chronic or even low-level exposure to Cd may potentially contribute to an increased likelihood of experiencing a fracture in the femoral neck [27]. Wang C et al., pointed out a negative association between blood Mn and femoral neck BMD was found in the fully adjusted model, especially for women aged 50–70 years [11]. In this study, after adjusting all potential confounding factors, we observed that only Cd level exposure was associated with femoral neck BMD, while no statistically significant differences were observed between other heavy metals and femoral neck BMD. The potential explanation is that the samples originate from diverse sources. The point worth noting is that, this study showed that mixture of heavy metal exposures was related to femoral neck BMD by using BKMR and qgcomp model. The findings also suggest a possible decrease in femoral neck BMD when individuals are simultaneously exposed to high levels of heavy metals.

Although the exact mechanism is unknown, these results help to reveal the harmful effect of mixed exposure to heavy metals on VCF risk. However, we also need to acknowledge some limitations of this study. First, due to a cross-sectional design in this study, the causal association between heavy metals and VCF is uncertain. More longitudinal studies are needed to confirm this finding. Second, we used the data of heavy metal was single-point measurements, which did not fully reflect the actual exposure levels of individuals [28]. Third, the nature of retrospective studies may introduce potential recall bias. Lastly, the NHANES database only is a representative of the United States population, and the results of this study were unlikely to be generalizable to other populations. More prospective epidemiological researches are required to confirm the current findings.

## Conclusion

In this analysis, no significant correlation was observed between co-exposure to Mn, Pb, Cd and Hg and VCF risk. But we found that an increase in the mixture of heavy metal exposures was associated with a decreased risk of femoral neck BMD. However, more prospective studies are needed to validate these findings and explore potential biological mechanisms.

## Supporting information

S1 Fig Individual metal exposures were grouped using Pearson correlation analysis. (PDF)

S2 Fig The exposure-response relationship of four heavy metals and ln-transformed VCF risk. (PDF)

S3 Fig The interaction between four heavy metals and ln-transformed VCF risk. (PDF)

S4 Fig The relationship of each additional quantile in the qgcomp index and the risk of VCF. (PDF)

S5 Fig Combined effects of four heavy metals mixtures and femoral neck BMD by BKMR analysis. (PDF)

S6 Fig Qgcomp model regression index weights of four heavy metals mixtures on femoral neck BMD. (PDF)

S1 Table The selection of covariates related to VCF or femoral neck BMD. (DOCX)

S2 Table Individual effects of four heavy metals on the femoral neck BMD. (DOCX)

S3 Table Qgcomp model to assess the joint effects of four heavy metals on the femoral neck BMD. (DOCX)
