# Supplementary figures and images for: Associations between co‑exposure to heavy metals and vertebral compression fracture, as well as femoral neck bone mineral density: A cross-sectional study from NHANES data
Source: PLoS One. 2024 May 22;19(5):e0303418. doi: 10.1371/journal.pone.0303418 (PMC11111051; doi:10.1371/journal.pone.0303418)

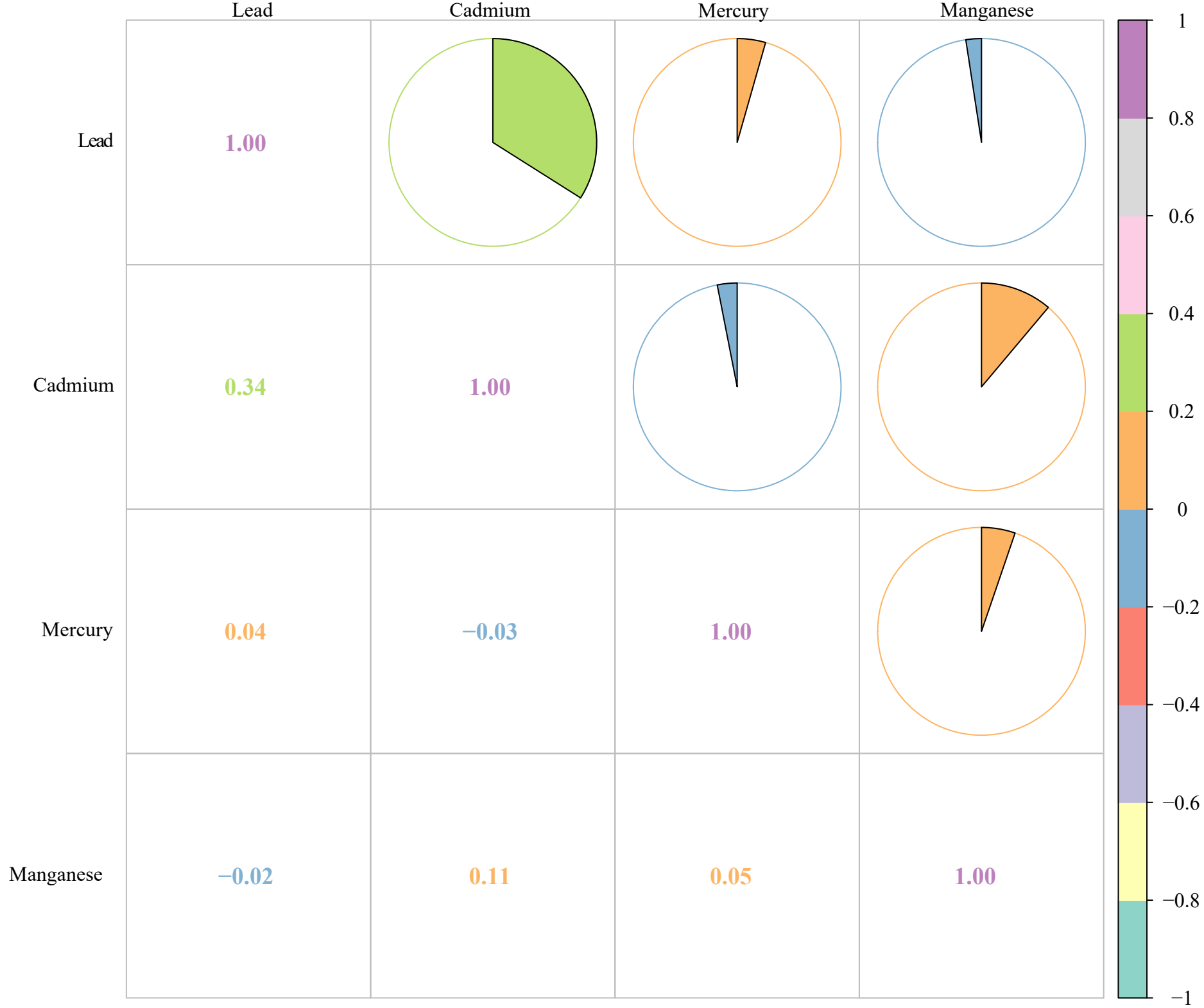

Supplement: S1 Fig — (PDF) [file pone.0303418.s001.pdf]

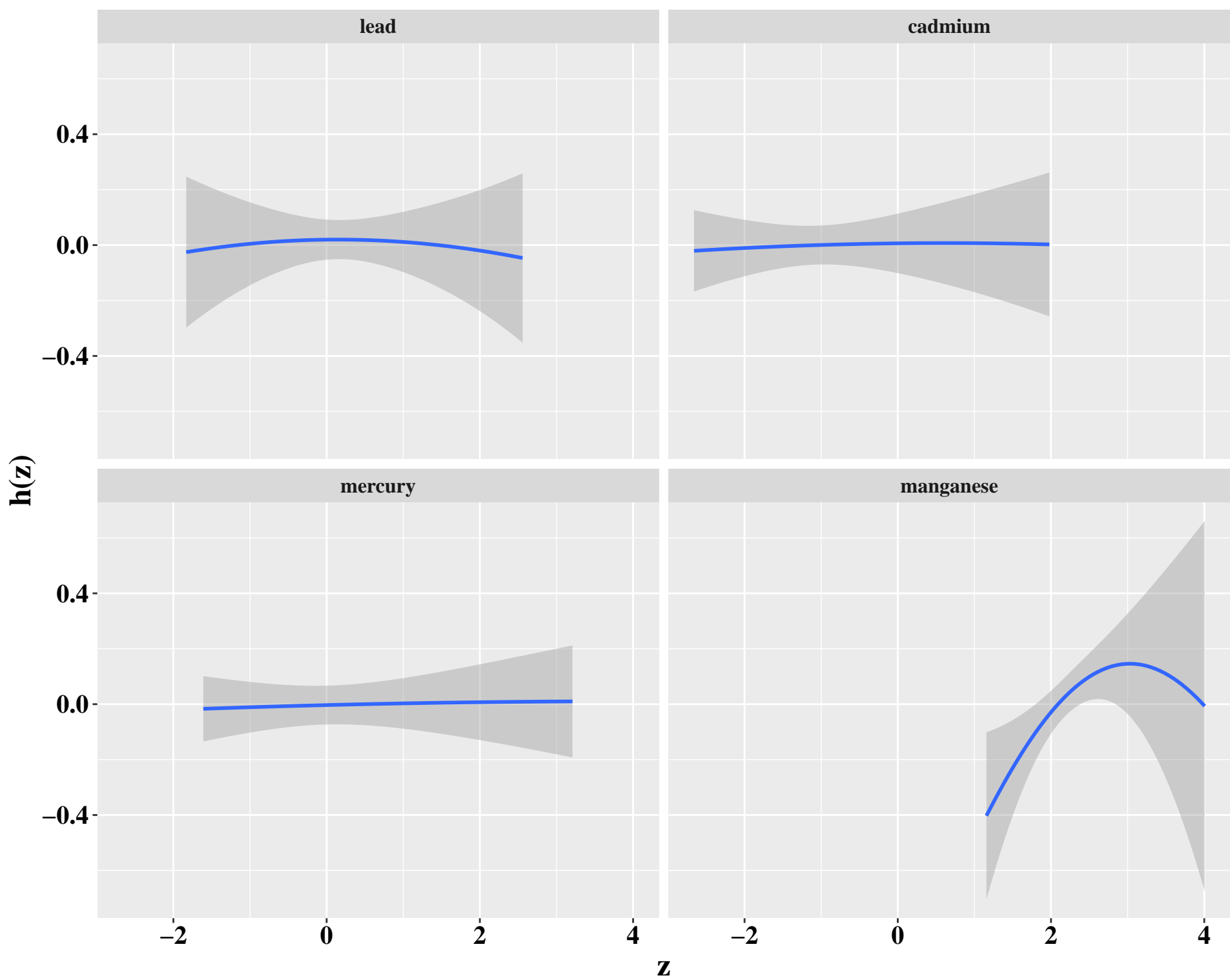

Supplement: S2 Fig — (PDF) [file pone.0303418.s002.pdf]

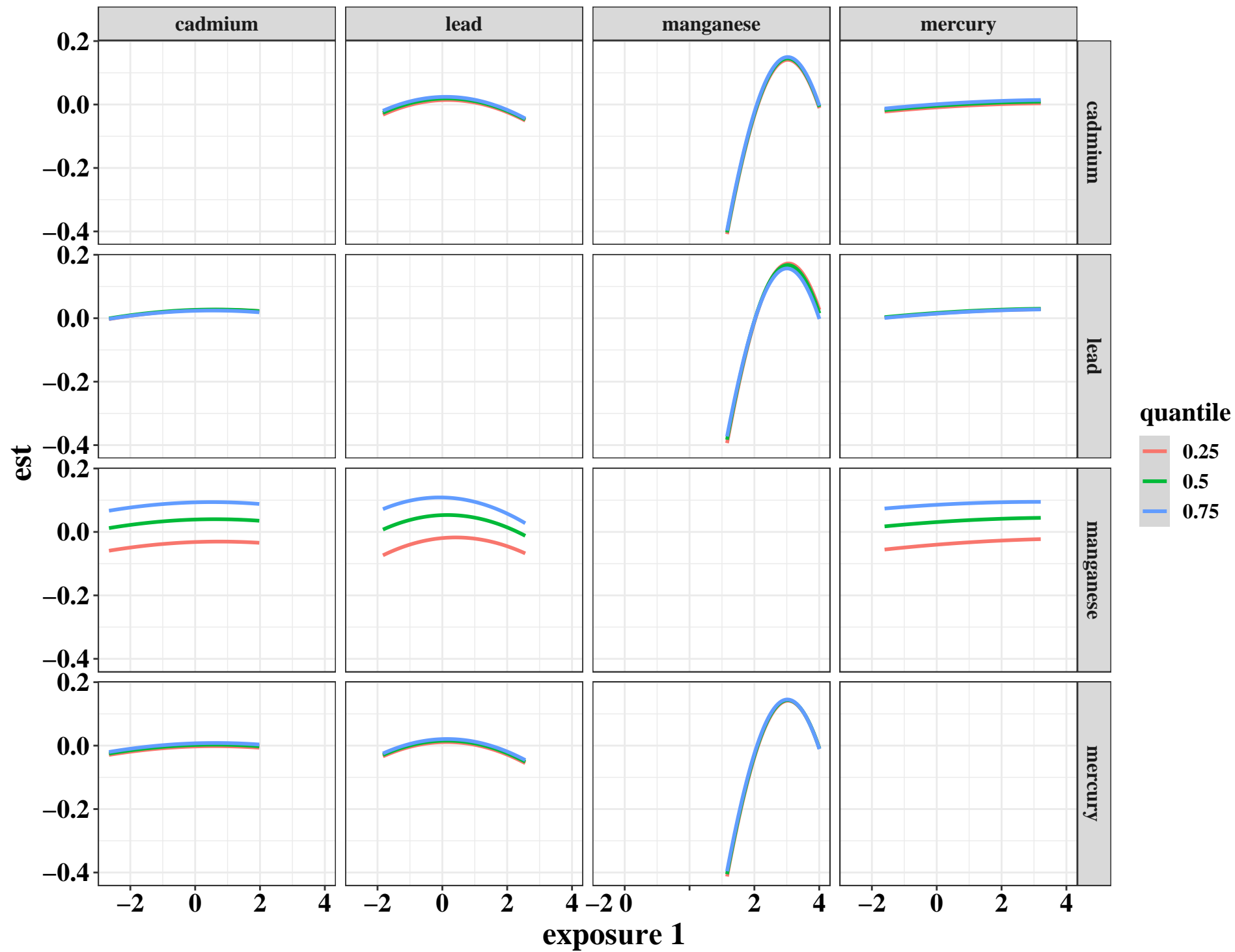

Supplement: S3 Fig — (PDF) [file pone.0303418.s003.pdf]

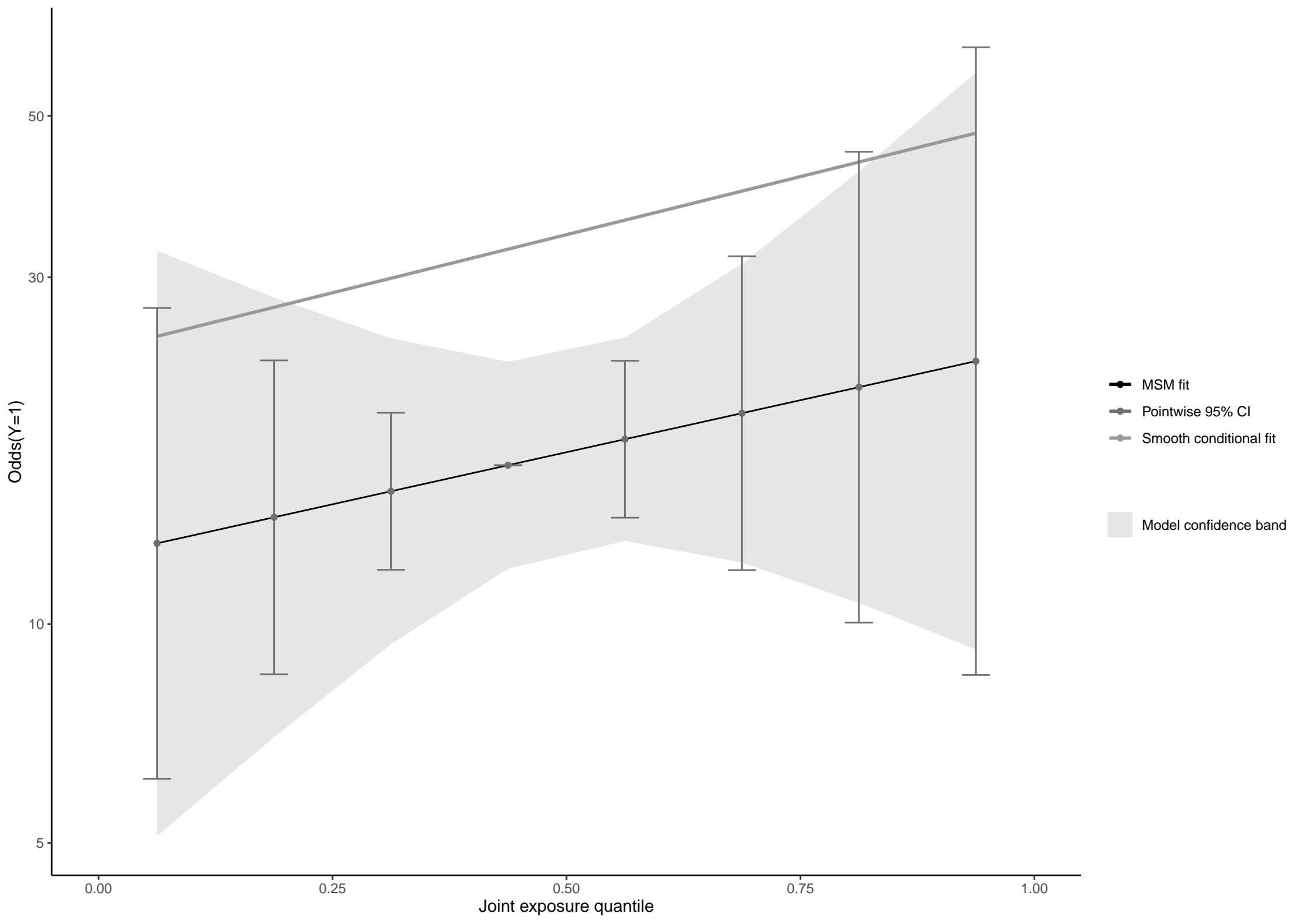

Supplement: S4 Fig — (PDF) [file pone.0303418.s004.pdf]

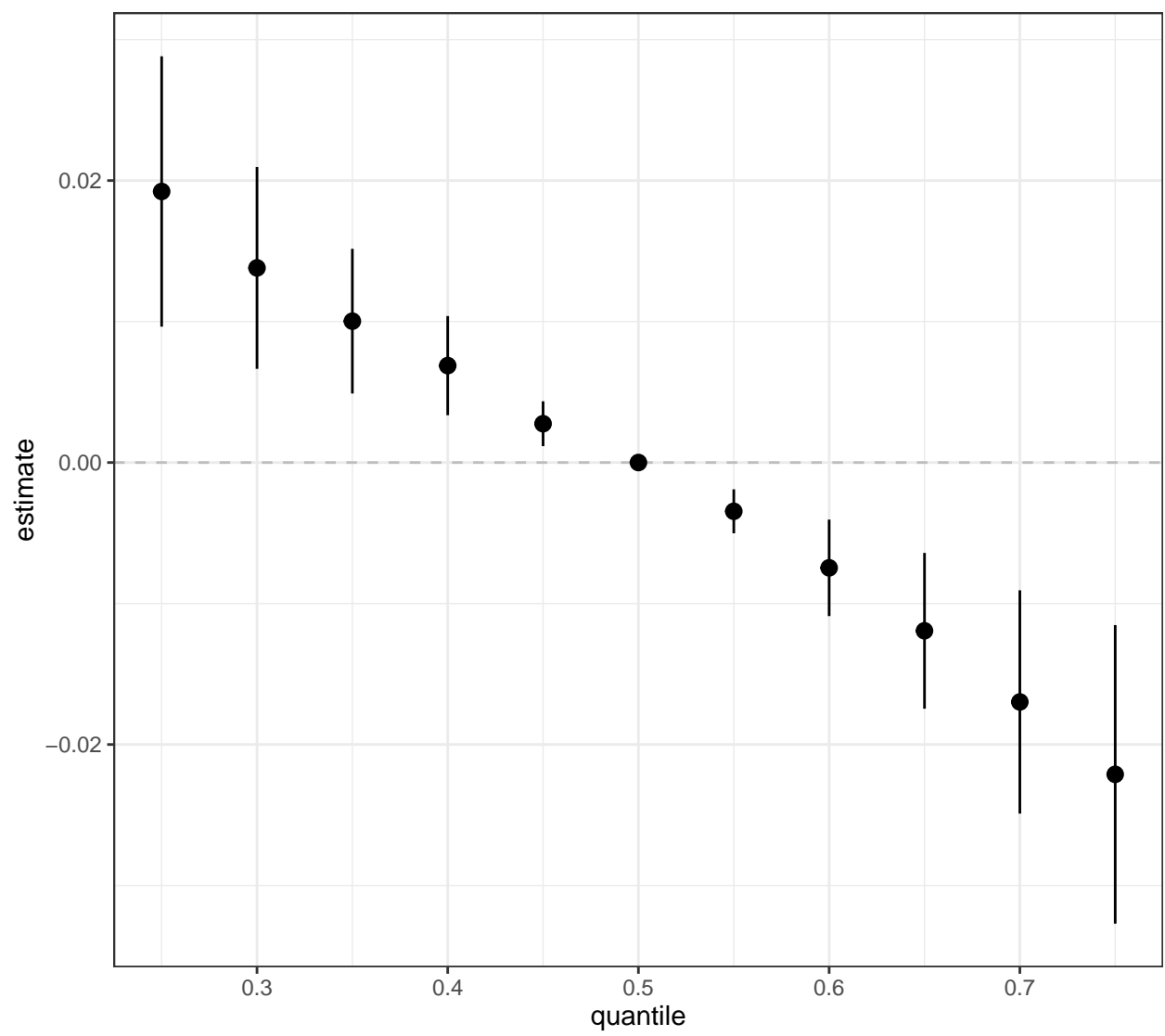

Supplement: S5 Fig — (PDF) [file pone.0303418.s005.pdf]

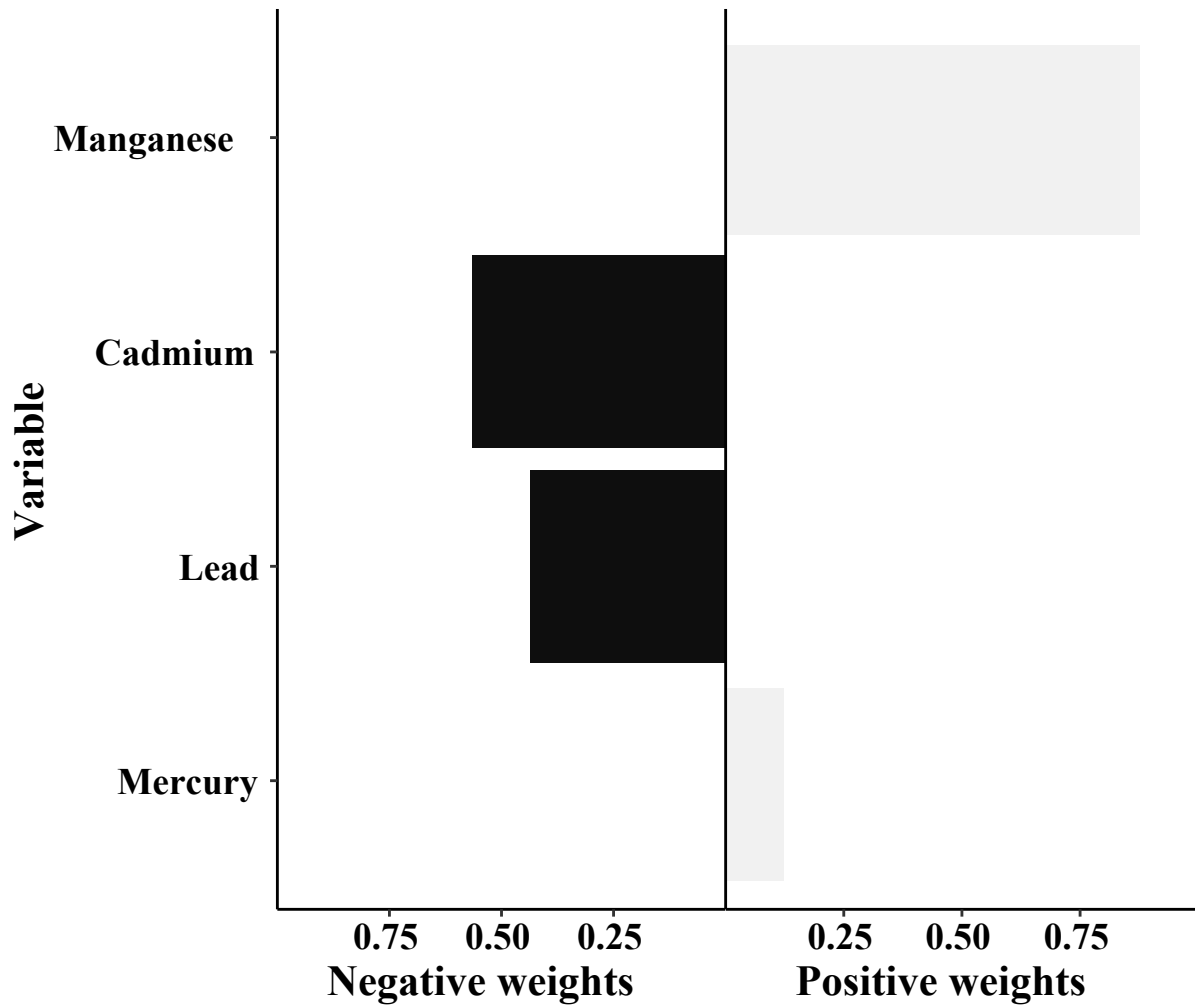

Supplement: S6 Fig — (PDF) [file pone.0303418.s006.pdf]
