# Supplementary material for: Associations between co‑exposure to heavy metals and vertebral compression fracture, as well as femoral neck bone mineral density: A cross-sectional study from NHANES data
Source: PLoS One. 2024 May 22;19(5):e0303418. doi: 10.1371/journal.pone.0303418 (PMC11111051; doi:10.1371/journal.pone.0303418)
Supplement: S1 Table — (DOCX) [file pone.0303418.s007.docx]

**Supplemental Table 1 The Selection of covariates related to VCF or femoral neck BMD**

| Variables | VCF | | Femoral neck BMD | |
| --- | --- | --- | --- | --- |
|  | OR (95%CI) | *P* | β (95% CI) | *P* |
| Age | 2.33 (1.48-3.68) | 0.001 | -0.00 (-0.01, -0.00) | <0.001 |
| Gender |  |  |  |  |
| Male | Ref |  | Ref |  |
| Female | 1.31 (0.59-2.87) | 0.481 | -0.06 (-0.08, -0.04) | <0.001 |
| Race/ethnicity |  |  |  |  |
| Mexican American | Ref |  | Ref |  |
| Other Hispanic | 1.60 (0.26-9.96) | 0.591 | -0.04 (-0.06, -0.02) | 0.002 |
| Non-Hispanic White | 2.47 (0.48-12.69) | 0.258 | -0.04 (-0.06, -0.02) | 0.001 |
| Non-Hispanic Black | 1.62 (0.34-7.66) | 0.517 | 0.07 (0.05, 0.10) | <0.001 |
| Other Race - Including Multi-Racial | 1.06 (0.18-6.16) | 0.941 | -0.05 (-0.08, -0.01) | 0.034 |
| Education |  |  |  |  |
| Less than 9th grade | Ref |  | Ref |  |
| 9-11th grade (Includes 12th grade with no diploma) | 1.37 (0.22-8.46) | 0.720 | 0.03 (-0.02, 0.07) | 0.276 |
| High school graduate/ GED or Equivalent | 1.49 (0.28-7.77) | 0.618 | 0.04 (0.01, 0.07) | 0.047 |
| Some College or AA degree | 0.98 (0.32-2.96) | 0.965 | 0.02 (-0.02, 0.05) | 0.340 |
| College Graduate or above | 0.84 (0.24-3.02) | 0.781 | 0.02 (-0.02, 0.07) | 0.314 |
| Marital status |  |  |  |  |
| Married | Ref |  | Ref |  |
| Not Married | 2.49 (1.26-4.90) | 0.012 | -0.04 (-0.06, -0.02) | 0.001 |
| Waist circumference | 0.84 (0.53-1.34) | 0.432 | 0.00 (0.00, 0.00) | <0.001 |
| BMI | 0.60 (0.39-0.94) | 0.029 | 0.01 (0.01, 0.01) | <0.001 |
| PIR | 0.73 (0.52-1.03) | 0.067 |  |  |
| Smoking |  |  |  |  |
| No | Ref |  | Ref |  |
| Yes | 0.79 (0.32-1.92) | 0.577 | -0.00 (-0.04, 0.03) | 0.791 |
| Quit smoking | 1.55 (0.59-4.08) | 0.252 | 0.00 (-0.03, 0.03) | 0.895 |
| Drinking |  |  |  |  |
| No | Ref |  | Ref |  |
| Yes | 0.98 (0.46-2.09) | 0.947 | 0.04 (0.02, 0.06) | 0.004 |
| Abstinence from alcohol | 2.52 (0.87-7.33) | 0.085 | 0.01 (-0.04, 0.05) | 0.792 |
| Physical activity | 0.81 (0.47-1.38) | 0.409 | 0.00 (-0.00, 0.00) | 0.075 |
| Parental fracture |  |  |  |  |
| No | Ref |  | Ref |  |
| Yes | 1.61 (0.46-5.64) | 0.431 | -0.06 (-0.09, -0.02) | 0.004 |
| Unknown | 1.44 (0.26-8.10) | 0.659 | -0.01 (-0.05, 0.03) | 0.635 |
| History of glucocorticoid use |  |  |  |  |
| No | Ref |  | Ref |  |
| Yes | 1.33 (0.55-3.21) | 0.501 | -0.04 (-0.07, -0.02) | 0.006 |
| History of anti-osteoporosis medication use |  |  |  |  |
| No | Ref |  |  |  |
| Yes | 6.42 (2.65-15.55) | <0.001 | -0.18 (-0.24, -0.13) | <0.001 |
| Diabetes |  |  |  |  |
| No | Ref |  | Ref |  |
| Yes | 0.97 (0.45-2.12) | 0.942 | -0.02 (-0.04, -0.01) | 0.025 |
| Hypertension |  |  |  |  |
| No | Ref |  | Ref |  |
| Yes | 2.50 (0.85-7.31) | 0.089 | -0.00 (-0.03, 0.02) | 0.684 |
| Total femur BMD | 0.29 (0.19-0.44) | <0.001 | - | - |
| Femoral neck BMD | 0.24 (0.16-0.36) | <0.001 | - | - |
| Menopause status |  |  |  |  |
| No | Ref |  | Ref |  |
| Yes | 4.77 (1.05-21.76) | 0.044 | -0.11 (-0.13, -0.08) | <0.001 |
| Not applicable (male) | 2.72 (0.44-16.89) | 0.261 | -0.01 (-0.04, 0.01) | 0.289 |
| Total energy intake | 0.69 (0.35-1.38) | 0.274 | 0.00 (0.00, 0.00) | 0.001 |
| Total calcium intake | 1.15 (0.83-1.60) | 0.381 | -0.00 (-0.00, 0.00) | 0.817 |
| Total Vitamin D intake | 0.91 (0.71-1.16) | 0.416 | -0.00 (-0.00, 0.00) | 0.445 |

VCF, vertebral compression fracture; GED, general educational development; AA, associate of arts; BMI, body mass index; PIR, poverty-to-income ratio; BMD, bone mineral density; OR, odds ratio; CI, confidence interval; Ref, reference.
