# Supplementary material for: Associations between co‑exposure to heavy metals and vertebral compression fracture, as well as femoral neck bone mineral density: A cross-sectional study from NHANES data
Source: PLoS One. 2024 May 22;19(5):e0303418. doi: 10.1371/journal.pone.0303418 (PMC11111051; doi:10.1371/journal.pone.0303418)
Supplement: S2 Table — (DOCX) [file pone.0303418.s008.docx]

**Supplemental Table 2 Individual effects of four heavy metals on the femoral neck BMD**

| Variables | Model 1 | | Model 2 | |
| --- | --- | --- | --- | --- |
|  | β (95% CI) | *P* | β (95% CI) | *P* |
| Pb | -0.01 (-0.02, -0.01) | 0.019 | -0.00 (-0.01, 0.00) | 0.321 |
| Cd | -0.03 (-0.05, -0.01) | 0.008 | -0.02 (-0.03, -0.01) | 0.043 |
| Hg | -0.00 (-0.01, 0.00) | 0.737 | 0.00 (-0.00, 0.01) | 0.674 |
| Mn | -0.00 (-0.00, 0.00) | 0.182 | -0.00 (-0.00, 0.00) | 0.971 |

Pb, lead; Cd, cadmium; Hg, mercury; Mn, manganese; CI, confidence interval; BMD, bone mineral density;

Model 1: did not adjust any variables;

Model 2: adjusted for age, gender, race/ethnicity, educational level, marital status, drinking, parental fracture, body mass index, waist circumference, history of glucocorticoid use, history of anti-osteoporosis medication use, diabetes, menopause status, and total energy.
