# Supplementary material for: Associations between co‑exposure to heavy metals and vertebral compression fracture, as well as femoral neck bone mineral density: A cross-sectional study from NHANES data
Source: PLoS One. 2024 May 22;19(5):e0303418. doi: 10.1371/journal.pone.0303418 (PMC11111051; doi:10.1371/journal.pone.0303418)
Supplement: S3 Table — (DOCX) [file pone.0303418.s009.docx]

**Supplemental Table 3 Qgcomp model to assess the joint effects of four heavy metals on the femoral neck BMD**

| Model | OR (95% CI) | *P* |
| --- | --- | --- |
| QGCOMP | 0.97 (0.95-0.98) | <0.001 |

CI, confidence interval; OR, odds ratio; Qgcomp, quantile-based g computation;

Qgcomp model adjusted for age, gender, race/ethnicity, educational level, marital status, drinking, parental fracture, body mass index, waist circumference, history of glucocorticoid use, history of anti-osteoporosis medication use, diabetes, menopause status, and total energy.
